# Short-and Long-Term Expression of Vegf: A Temporal Regulation of a Key Factor in Diabetic Retinopathy

**DOI:** 10.3389/fphar.2021.707909

**Published:** 2021-08-16

**Authors:** Claudio Bucolo, Annalisa Barbieri, Ilaria Viganò, Nicoletta Marchesi, Francesco Bandello, Filippo Drago, Stefano Govoni, Gianpaolo Zerbini, Alessia Pascale

**Affiliations:** ^1^ Department of Biomedical and Biotechnological Sciences, School of Medicine, University of Catania, Catania, Italy; ^2^ Center for Research in Ocular Pharmacology–CERFO, University of Catania, Catania, Italy; ^3^ Department of Drug Sciences, Pharmacology Section, University of Pavia, Pavia, Italy; ^4^ Complications of Diabetes Unit, Diabetes Research Institute, IRCCS Ospedale San Raffaele, Milan, Italy; ^5^ Department of Ophthalmology, IRCCS Ospedale San Raffaele, Vita-Salute University, Milan, Italy

**Keywords:** VEGF, ELAV/HuR, retinopathy, diabetes, hyperglycemia, akita mouse

## Abstract

To investigate the role of vascular endothelial growth factor (VEGF) at different phases of diabetic retinopathy (DR), we assessed the retinal protein expression of VEGF-A_164_ (corresponding to the VEGF_165_ isoform present in humans, which is the predominant member implicated in vascular hyperpermeability and proliferation), HIF-1α and PKCβ/HuR pathway in *Ins2* ^*Akita*^ (diabetic) mice at different ages. We used C57BL6J mice (WT) at different ages as control. Retina status, in terms of tissue morphology and neovascularization, was monitored *in vivo* at different time points by optical coherence tomography (OCT) and fluorescein angiography (FA), respectively. The results showed that VEGF-A_164_ protein expression increased along time to become significantly elevated (*p* < 0.05) at 9 and 46 weeks of age compared to WT mice. The HIF-1α protein level was significantly (*p* < 0.05) increased at 9 weeks of age, while PKCβII and HuR protein levels were increased at 46 weeks of age compared to WT mice. The thickness of retinal nerve fiber layer as measured by OCT was decreased in *Ins2* ^*Akita*^ mice at 9 and 46 weeks of age, while no difference in the retinal vasculature were observed by FA. The present findings show that the retina of the diabetic *Ins2* ^*Akita*^ mice, as expected for mice, does not develop proliferative retinopathy even after 46 weeks. However, diabetic *Ins2* ^*Akita*^ mice recapitulate the same evolution of patients with DR in terms of both retinal neurodegeneration and pro-angiogenic shift, this latter indicated by the progressive protein expression of the pro-angiogenic isoform VEGF-A_164,_ which can be sustained by the PKCβII/HuR pathway acting at post-transcriptional level. In agreement with this last concept, this rise in VEGF-A_164_ protein is not paralleled by an increment of the corresponding transcript. Nevertheless, the observed increase in HIF-1α at 9 weeks indicates that this transcription factor may favor, in the early phase of the disease, the transcription of other isoforms, possibly neuroprotective, in the attempt to counteract the neurodegenerative effects of VEGF-A_164._ The time-dependent VEGF-A_164_ expression in the retina of diabetic *Ins2* ^*Akita*^ mice suggests that pharmacological intervention in DR might be chosen, among other reasons, on the basis of the specific stages of the pathology in order to pursue the best clinical outcome.

## Introduction

Diabetic retinopathy (DR) is the major eye complication of diabetes mellitus and represents the leading cause of preventable blindness in the working age population, where roughly 90% of patients with type 1 diabetes and approximately 80% with type 2 diabetes for over 10 years will face the disease (Campbell and Doyle, 2019). Among common causes for blindness or severe vision impairment, DR takes the fifth place, and a recent meta-analysis underscores that, due to population growth together with a rise in the corresponding average age, the age-standardized prevalence of DR-related blindness will increase in the future (Leasher et al., 2016; Hammes 2018). The mechanisms involved in the progression of DR, characterized by an initial and prolonged ischemic phase followed by an aggressive vascular proliferation, are still argument of investigation. In particular, the role of VEGF (a factor involved in angiogenesis and cell permeability) on DR progression is still poorly understood. According to the level of microvascular- and ischemic-related damage, DR can be classified into two stages: an early non-proliferative stage (NPDR) and an advanced proliferative stage (PDR). The earliest sign of DR is the loss of pericytes contributing to inner blood-retinal barrier breakdown (Arboleda-Velasquez et al., 2015). Hallmarks of DR are also the increasing thickness of the basement membrane, the hyper-permeability, and the formation of microaneurysms. These functional alterations are followed by microvascular occlusions leading to a progressive retinal ischemia that induces the expression of the Vascular Endothelial Growth Factor-A (VEGF-A). VEGF-A, and especially the main pro-angiogenic isoform VEGF-A_165_, has a primary role in promoting vascular hyperpermeability; indeed, *via* phosphorylation of endothelial tight junction proteins it modulates their degradation, finally leading to blood-retinal barrier disruption (Moran et al., 2016). Capillary leakage and subsequent retinal exudation and edema are typical features of the disease. Further, VEGF-A_165_ is a potent mitogen for endothelial cells triggering their proliferation, migration and tube formation resulting in the growth of new blood vessels along the inside surface of the retina and in the vitreous that, however, in the diabetic retina are fragile and may break (Antonetti et al., 2012). In turn, these events may entail vitreous hemorrhage, subsequent fibrosis, and tractional retinal detachment with risk of permanent vision loss in the affected eye (Dulull et al., 2019). Hence, due to this dual capability to promote both vascular permeability and pathologic angiogenic proliferation, VEGF-A constitutes a key player in DR and therefore a compelling druggable target (Amadio et al., 2016a; Zhao and Singh, 2018). Incidentally, analysis of VEGF-A interaction with binding domains of anti-angiogenic agents used in clinical practice is crucial in order to improve the design of new drugs (Platania et al., 2015).

VEGF-A expression can be regulated at both transcriptional, *via* hypoxia inducible factor-1α (HIF-1α), and post-transcriptional level through the PKCβ/HuR cascade (Amadio et al., 2008; Amadio et al., 2010; Amadio et al., 2012). Inside the retina the cells that may show an increased expression of VEGF in case of DR are retinal pigmented epithelial cells, pericytes, astrocytes, müller cells, glial cells, and endothelial cells (Antonetti et al., 2012).

The aim of the present study was to investigate the role of the PKCβII/HuR/VEGF-A pathway in the development of DR using the *Ins2* ^*Akita*^ mouse animal model, which is characterized by a dominant mutation that induces the development of a spontaneous insulin-dependent diabetes with a rapid onset (Barber et al., 2005). Moreover, besides assessing the post-transcriptional involvement of the PKCβII/HuR cascade in regulating VEGF-A_164_ (corresponding to the VEGF_165_ isoform present in humans) content, we also examined, in *Ins2* ^*Akita*^ mice at different ages, the expression of HIF-1α to figure out the contribution of this specific transcriptional factor in the regulation of VEGF-A_164_ expression.

## Materials and Methods

### Animals

The recent identification of *Ins2* ^*Akita*^ mouse where diabetes, followed along time by the appearance of early signs of DR, develops as a consequence of a spontaneous mutation of the Insulin 2 gene makes it a unique model of “human diabetic complications” suitable for testing novel preventive approaches (Barber et al., 2005; Han et al., 2013).

Two groups of animals (5 per group), diabetic *Ins2* ^*Akita*^ and normoglycemic (C57BL6) mice were followed for 46 weeks. Only males have been included in this study because disease progression in females is slower and less uniform (Han et al., 2013). *Ins2* ^*Akita*^ female mice are resistant to develop diabetes and glycemia is often slightly, but not significantly increased when compared to controls (Al-Awar et al., 2016). Male C57BL6 and *Ins2* ^*Akita*^ mice were housed in cages in a temperature-controlled room with a 12:12 light–dark cycle and free access to food and tap water. Body weight (g) and blood glucose concentrations (mg/dl) were measured weekly during the experimental period. The experimental study was approved by the Institutional Animal Care and Use Committee (IACUC) of the San Raffaele Scientific Institute in Milan, according to the National Legislation (D.L. 116/1992) and the European Directive (2010/63/EU) about the use of laboratory animals, and with the license of the Italian Board of Health.

### Preparation of the Samples and Western Blotting

Retinae were homogenized, using a Teflon/glass homogenizer, in the following buffer: 20 mM Tris (pH 7.4), 2 mM EDTA, 0.5 mM EGTA, 50 mM β-mercaptoethanol, 0.32 mM sucrose and a protease inhibitor cocktail (Roche Molecular Biochemicals, Mannheim, Germany) at the dilution suggested by the manufacturer. Proteins were measured according to Bradford’s method, using bovine albumin as internal standard. Then, the proteins were diluted in Sodium Dodecyl Sulphate (SDS) protein gel loading solution, boiled for 5 min, separated on SDS-PolyAcrylamide Gel Electrophoresis, and processed following standard procedures. The mouse monoclonal anti-ELAV/HuR antibody (Santa Cruz Biotech. Inc., Dallas, TX, United States) was diluted at 1:1,000. The rabbit monoclonal antibodies anti-VEGF-A (Abcam, United Kingdom) and anti-HIF1α (Cell Signalling Technology, Netherlands) were diluted at 1:750 and 1:1,000, respectively. The rabbit polyclonal PKC βII (Santa Cruz Biotech. Inc.) was diluted at 1: 500, while the rat monoclonal antibody anti-α-tubulin (Thermo Fisher Scientific, Waltham, MA, United States) was diluted at 1:1,000. The specific antibodies were diluted in TBST buffer [10 mM Tris-HCl, 100 mM NaCl, 0.1% (v/v) Tween 20, pH 7.5] containing 6% milk. The nitrocellulose membrane signals were detected by chemiluminescence. Experiments were performed at least three times for each tissue preparation; the same membranes were reprobed with α-tubulin antibody to normalize the data. Statistical analysis of western blot data was performed on the densitometric values obtained with the ImageJ 1.50i software (downloadable at http://imagej.nih.image/ij).

### Real Time RT-PCR

RNA was extracted from total homogenates by using RNeasy Mini Kit (Qiagen, Germany). The reverse transcription was performed following standard procedures. PCR amplifications were carried out using the Rotor-Gene Q instrument (Qiagen) in the presence of QuantiTect SYBR Green PCR mix (Qiagen) with the specific primers for VEGF_164_ (provided by SIGMA). The GAPDH mRNA was chosen as the reference gene to normalize the data (the specific primers were provided by Qiagen).

### Optical Coherence Tomography and Fluorescein Angiography

Optical coherence tomography (OCT) and fluorescein angiography (FA) were performed as previously described (Buccarello et al., 2017), taking advantage of the Micron IV instrument (Phoenix Research Laboratories, Pleasanton, CA, United States). Briefly, after anesthesia, mydriasis was induced by administering a drop of tropicamide 0.5% (Visumidriatic, Tibilux Pharma, Milan, Italy) in each eye. OCT images were acquired by performing a circular scan of 550 μm of diameter around the optic nerve head. Both eyes were examined, and the results were averaged. The segmentation of retinal layers was performed using Insight software (Phoenix Research Laboratories, Pleasanton, CA, United States), OCT was followed by the FA study. A solution of 1% fluorescein (5 ml/kg Monico S.p.A., Venezia, Italy) was administered by a single intraperitoneal injection (100 μL). For each animal, the images of central and peripheral retinal vasculature were acquired.

### Statistical Analysis

For statistical analysis, the GraphPad Instat statistical package (version 3.05 GraphPad software, San Diego, CA, United States) was used. The data were analyzed by analysis of variance (ANOVA) followed, when significant, by an appropriate *post hoc* comparison test, as detailed in the legends. Differences were considered statistically significant when *p* values <0.05.

## Results

**FIGURE 1:**
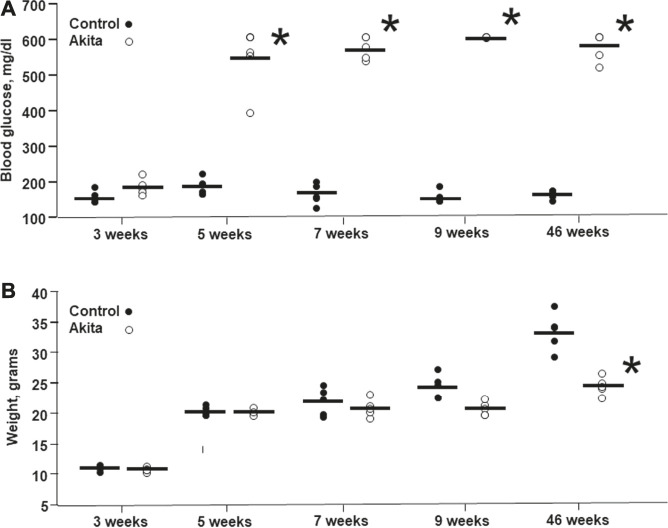
Blood glucose levels and body weight in wild-type and *Ins2* ^*Akita*^ mice animals at different ages. Blood glucose levels **(A)** are expressed in mg/dL, while body weight **(B)** is expressed in grams. The values are shown as filled dots for wild-type mice and open dots for *Ins2* ^*Akita*^ animals *p < 0.05 student t-test, *vs* wild-type.

Blood glucose levels, measured at 5, 7, 9, and 46 weeks of age, were significantly increased in *Ins2* ^*Akita*^ mice in comparison with their relative wild-type littermates (Figure 1A). Conversely, body weight measured at 9 and 46 weeks of age was significantly reduced in *Ins2* ^*Akita*^ mice, as a consequence of glycosuria, in comparison with their respective wild-type controls, reaching a statistical significance in 46 weeks old animals (Figure 1B).

VEGF protein is increased in *Ins2* ^*Akita*^ mice: a time-dependent contribution of transcriptional and post-transcriptional mechanisms.

**FIGURE 2:**
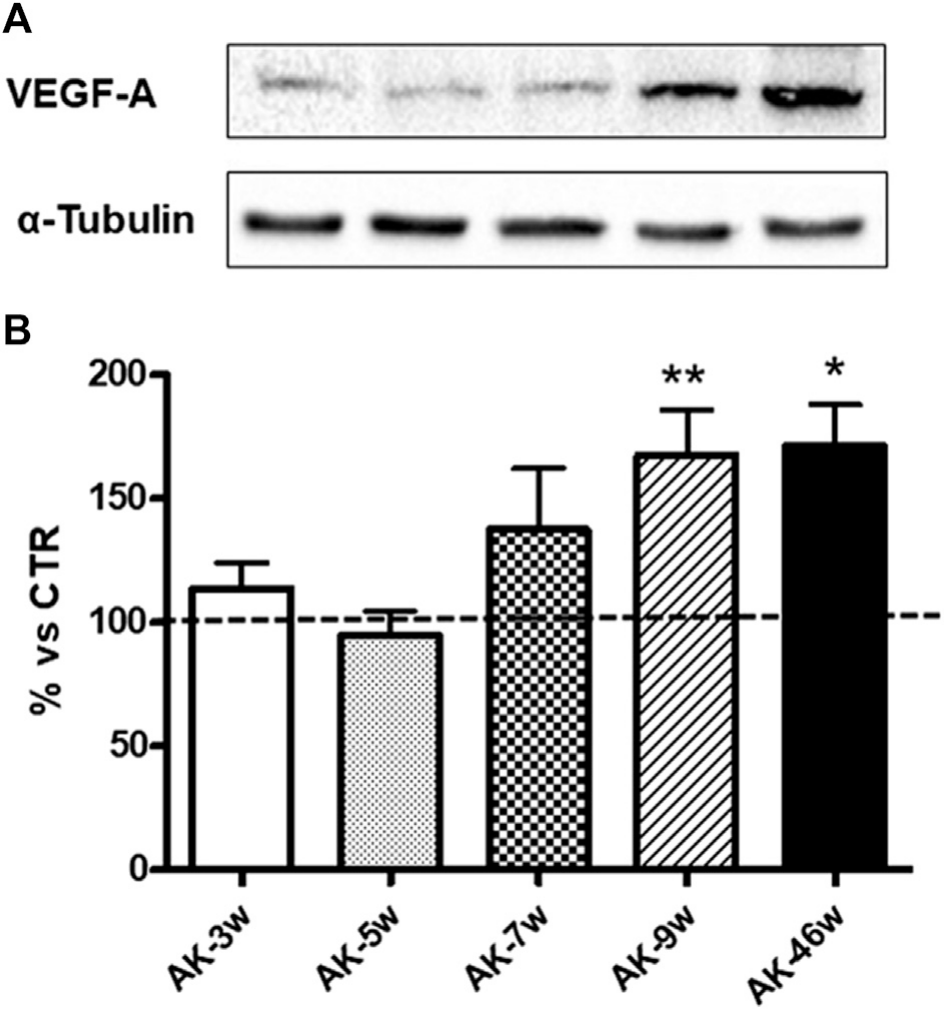
VEGF-A protein levels in *Ins2* ^*Akita*^ mice at different ages. **(A)**: Representative western blottings of VEGF-A_164_ and the respective *α*-tubulin, measured in the same samples, of total homogenates of retinae from *Ins2* ^*Akita*^ (AK) mice at different ages (w = weeks). **(B)**: Densitometric analysis of VEGF-A_164_ immunoreactivities in the total homogenates of retinae from wild-type and AK mice at different ages. Alpha-tubulin was used as a loading control. Results are expressed as % (±S.E.M.) *vs* control wild-type (100%, dashed line). *p < 0.05, **p < 0.01 *v*s wild-type; Tukey’s Multiple Comparison *post-hoc* test, n = 8–10.

**FIGURE 3:**
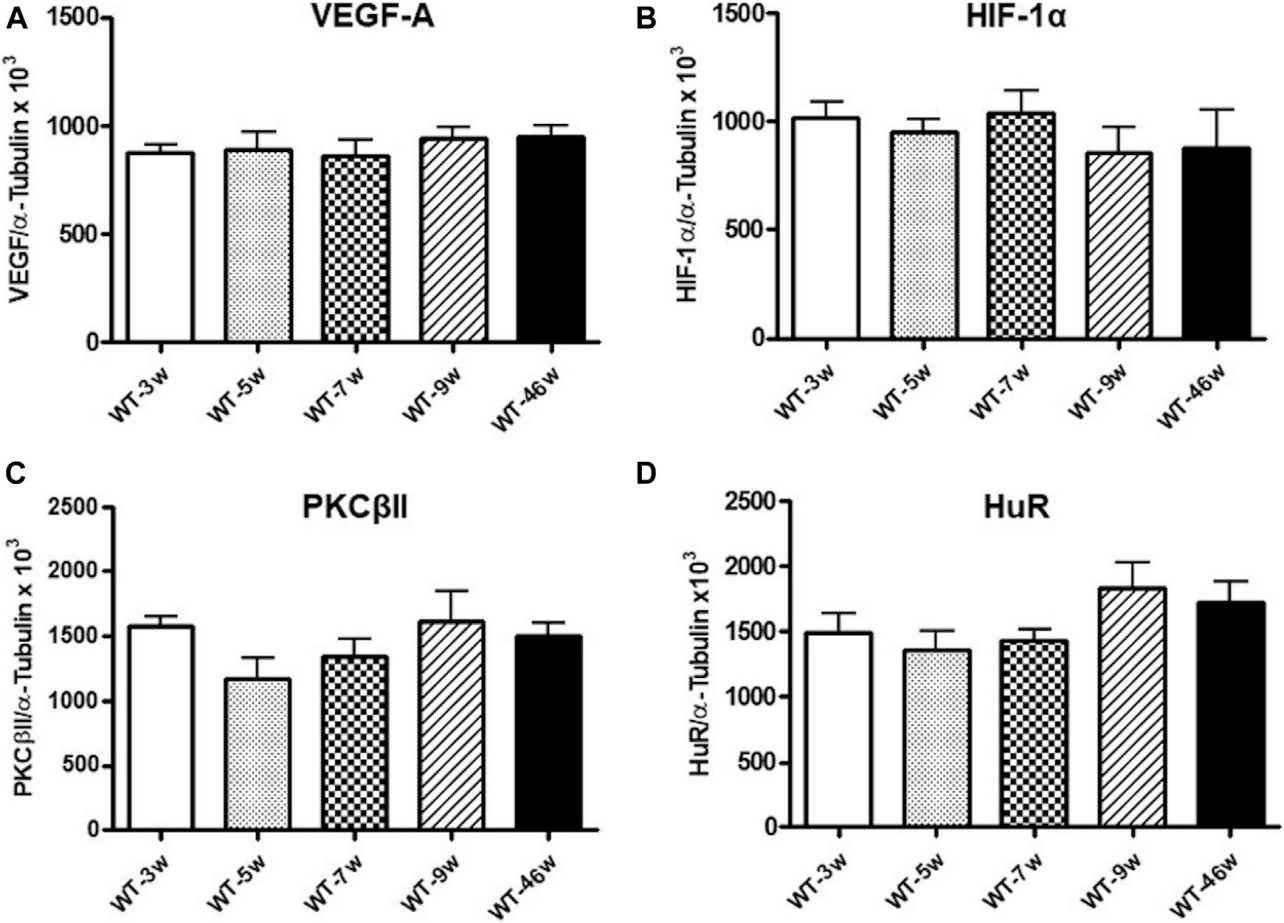
VEGF-A, HIF-1α, PKCβII and HuR protein levels in wild-type mice at different ages. Densitometric analysis of VEGF-A_164_ **(A)**, HIF-1α **(B)**, PKCβII **(C)**, and HuR **(D)** immunoreactivities in the total homogenates of retinae from wild-type (WT) mice at different ages (w = weeks). Alpha-tubulin was used as a loading control. Results are expressed as mean grey levels ratios x 10^3^ (mean ± S.E.M.) of VEGF-A_164_/α-tubulin **(A)**, HIF-1α/α-tubulin **(B)**, PKCβII/α-tubulin **(C)** and HuR/α-tubulin **(D)** immunoreactivities measured by Western blotting, n = 5.

Given the key role of VEGF-A_165_ in DR development, we assessed its protein expression in wild-type and *Ins2* ^*Akita*^ mice at different ages. As shown in Figure 2, in *Ins2* ^*Akita*^ mice we found an increase in VEGF-A_164_ content (as previously mentioned, this isoform corresponds to the VEGF-A_165_ present in humans) already at 7 weeks (+38%), which reaches a statistical significance in 9 (+67%) and 46 (+71%) weeks old animals with respect to their relative wild-type littermates. No age-dependent changes in VEGF-A_164_ protein basal levels, measured in an independent set of experiments, were observed among wild-type mice (Figure 3).

We also measured VEGF-A_164_ mRNA levels *via* Real-time PCR. Preliminary results indicate a significant decrease in VEGF-A_164_ transcript content at 9 and 46 weeks (−78%, *p* < 0.005 and −63%, *p* < 0.05, respectively) compared to 3 weeks *Ins2* ^*Akita*^ mice.

With the purpose of dissecting the implication of both transcriptional and post-transcriptional mechanisms in the regulation of VEGF-A_164_ protein expression, we measured, respectively, HIF-1α and PKCβII/HuR protein levels in the retina from wild-type and *Ins2* ^*Akita*^ mice at different ages.

**FIGURE 4:**
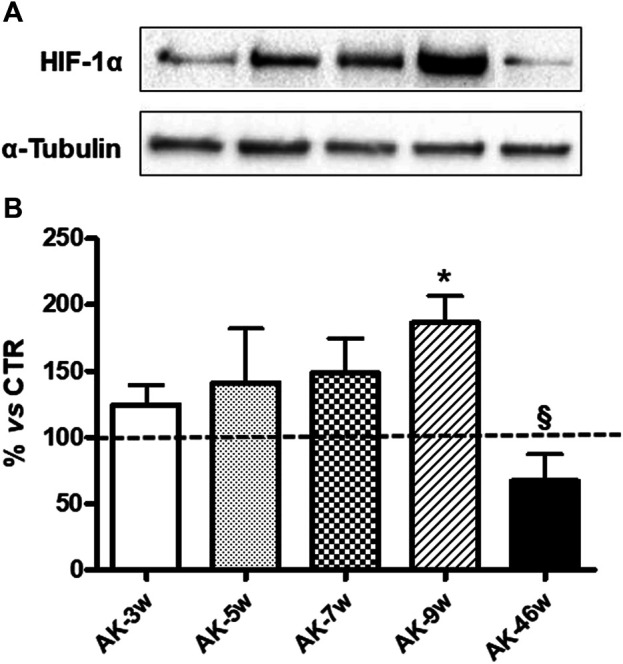
HIF-1α protein levels in *Ins2* ^*Akita*^ mice at different ages. **(A)**: Representative western blottings of HIF-1α and the respective α-tubulin, measured in the same samples, of total homogenates of retinae from *Ins2* ^*Akita*^ (AK) mice at different ages (w = weeks). **(B)**: Densitometric analysis of HIF-1α immunoreactivities in the total homogenates of retinae from wild-type and AK mice at different ages. Alpha-tubulin was used as a loading control. Results are expressed as % (±S.E.M.) *vs* control wild-type (100%, dashed line). *p < 0.05 *vs* wild-type and ^§^ *p* < 0.05 *vs* AK-9w; Tukey’s Multiple Comparison *post-hoc* test, n = 8–10.

Concerning HIF-1α, as depicted in Figure 4, in *Ins2* ^*Akita*^ mice we observed a progressive increase in its protein content starting from 3 weeks (+25%) to 7 weeks old animals (5 weeks: +41%; 7 weeks: +49%), reaching a statistically significant rise in 9 weeks old mice (+87%). Instead, a decrease was found in 46 weeks old *Ins2* ^*Akita*^ mice (−33%), whose levels were significantly lower with respect to 9 weeks old *Ins2* ^*Akita*^animals. No age-dependent changes in HIF-1α protein basal levels, measured in an independent set of experiments, were observed among wild-type mice (Figure 3).

**FIGURE 5:**
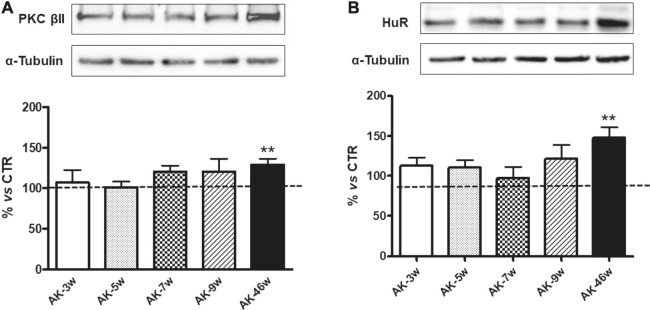
PKCβII and HuR protein levels in *Ins2* ^*Akita*^ mice at different ages. Upper panels: Representative western blottings of PKCβII **(A)**, HuR **(B)** and the respective *α*-tubulin, measured in the same samples, of total homogenates of retinae from *Ins2* ^*Akita*^ (AK) mice at different ages (w = weeks). Lower panels: Densitometric analysis of PKCβII **(A)** and HuR **(B)** immunoreactivities in the total homogenates of retinae from wild-type and AK mice at different ages. Alpha-tubulin was used as a loading control. Results are expressed as % (±S.E.M.) *vs* control wild-type (100%, dashed line). **p < 0.01 *vs* wild-type; unpaired *t*-test, n = 8–10.

We then investigated the PKCβII/HuR pathway, since we previously demonstrated, in another animal model of DR, its key involvement in the post-transcriptional control of VEGF-A protein expression (Amadio et al., 2010). As shown in Figure 5, we observed a significant increase in both PKCβII (+29%) and HuR (+48%) protein levels only in 46 weeks old *Ins2* ^*Akita*^ mice. No age-dependent changes in PKCβII and HuR protein basal levels, measured in an independent set of experiments, were observed among wild-type mice (Figure 3).

### OCT and Fluorescein Angiography Evaluation

**FIGURE 6:**
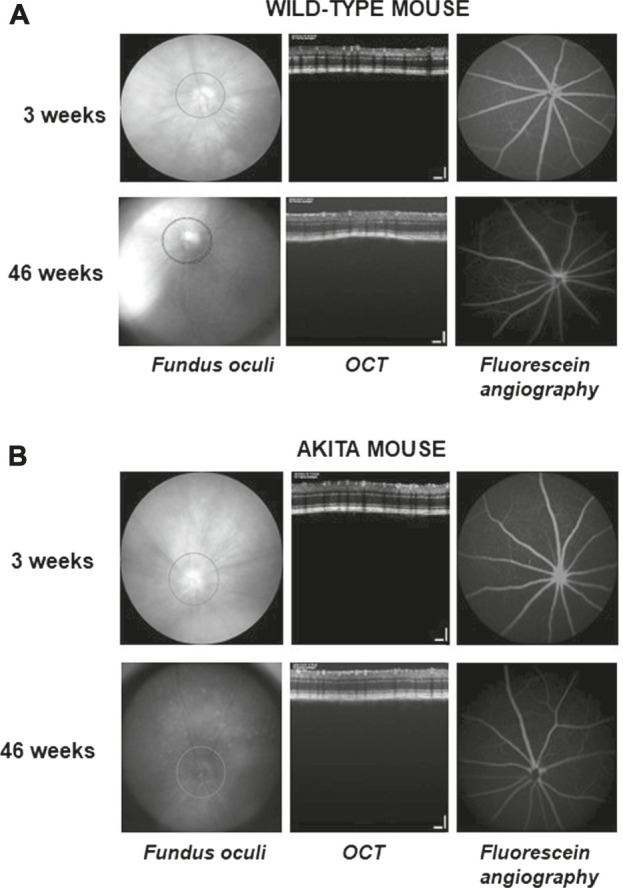
Fundus oculi **(left)**, optical coherence tomography (OCT; center) and fluorescein angiography **(right)** performed during the study in wild-type and *Ins2* ^*Akita*^ animals. The shown images were taken in the left eye of a representative wild-type **(A)** or *Ins2* ^*Akita*^ **(B)** mice at 3 and 46 weeks of age.

**FIGURE 7:**
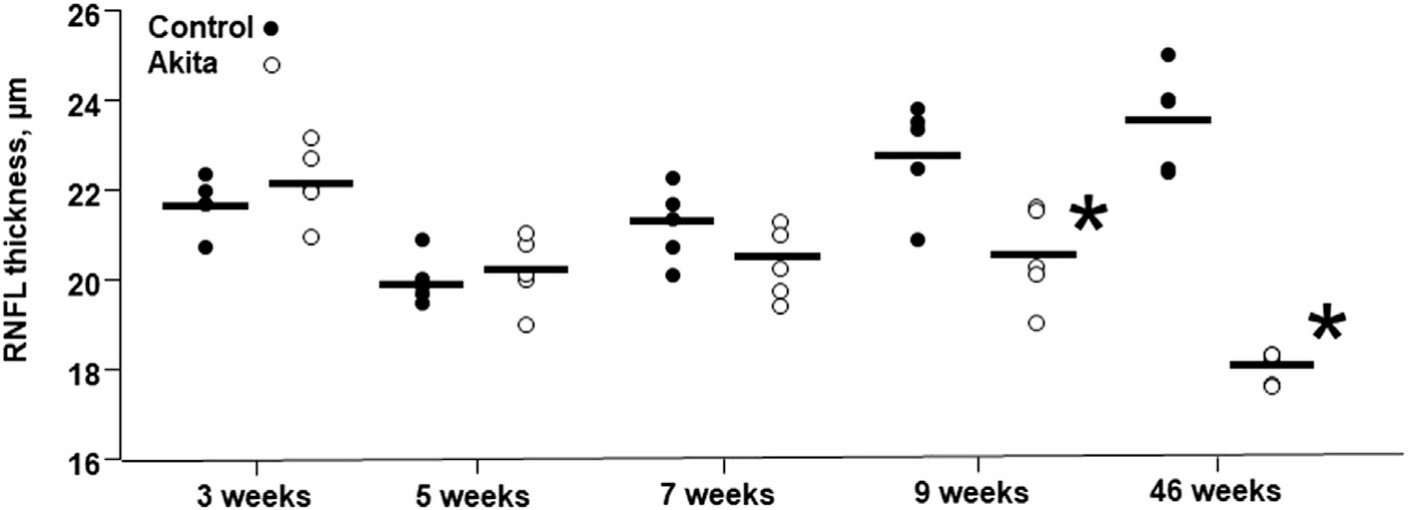
Retinal nerve fiber layer thickness in wild-type and *Ins2* ^*Akita*^ animals. Retinal nerve fiber layer (RNFL) thickness is expressed in µm and the values are shown as filled dots for wild-type mice and open dots for *Ins2* ^*Akita*^ animals. *p < 0.05, student *t*-test *vs* control wild-type.

OCT and fluorescein angiography were performed during the entire study in wild-type and *Ins2* ^Akita^ diabetic mice (Figure 6). The collected images did not highlight any retinal signs of vascular dysfunction in both groups of animals. Thickness of Retinal Nerve Fiber Layer (RNFL) was also examined. The results show a significant decrease in RNFL thickness in 9 weeks of age *Ins2* ^*Akita*^ mice compared to wild-type littermates, which becomes even more pronounced in 46 weeks old animals (Figure 7).

## Discussion

Diabetic retinopathy is the primary cause of blindness in adults living in industrialized countries, being the global prevalence around 30–35% within the diabetic population. The loss of visual function is mainly associated with macular edema and the proliferative stage of the disease, with a dramatic impact in terms of countries health system costs. DR is triggered by the chronic hyperglycemia linked to the diabetic condition and by the following metabolic stress. This altered milieu induces changes at microvascular level that result in the inability of capillaries to guarantee to the retina the proper blood supply, thus entailing the formation of non-perfused areas and the development of a hypoxic environment that promotes the production of VEGF-A, a pivotal player in DR pathophysiology (Rigo et al., 2020).

VEGF-A belongs to a family that also includes VEGF-B, -C, -D, and placental growth factor (Amadio et al., 2016a; Ferrara and Adamis, 2016). The human VEGF-A gene consists of eight exons separated by seven introns determining the generation of different isoforms, being the VEGF-A_165_ the predominant member and the main isoform implicated in vascular hyperpermeability and proliferation (Amadio et al., 2016a). In the diabetic retina, the expression of VEGF-A can be regulated by different pathways, including Protein Kinase C (PKC) (Clarke and Dodson, 2007; Yokota et al., 2007; Ye et al., 2010). PKC consists of at least 10 serine-threonine kinases ubiquitously expressed and involved in several cellular functions (Battaini and Mochly-Rosen, 2007; Govoni et al., 2010). It is worth of note, that the diabetes-related hyperglycemia induces a rise in the amount of diacylglycerol, the physiological activator of PKC. Among the various isoforms, the PKCβ seems to be the isoenzyme primarily activated in the retina, although other PKCs can be also implicated (Aiello, 2002; Kim et al., 2010).

In the present study, we used the *Ins2* ^*Akita*^ mouse as an animal model of type 1 diabetes, which is endowed with a dominant mutation that induces the development of a spontaneous insulin-dependent diabetes with a rapid onset (Barber et al., 2005). Notably, the mutation in the Insulin 2 gene elicits a conformational change in the insulin protein and its consequent accumulation in pancreatic β cells, leading to β-cells death (Olivares et al., 2017). As supported by literature data, the *Ins2* ^*Akita*^ mouse is an excellent model to explore the molecular mechanisms implicated in the initiation and early progression of DR. The development, in the *Ins2* ^*Akita*^ mouse of the late, neovascular stages of DR remains presently unclear (Han et al., 2013; McLenachan et al., 2013). Indeed, we showed that by 5 weeks of age the animals present already significantly elevated levels of blood glucose compared to wild-type littermates, while a decrease in the body weight, due to glycosuria, is evident starting from 9 weeks of age. We also observed a time-dependent increase in VEGF-A_164_ (this isoform corresponds to the VEGF-A_165_ present in humans) content at retinal level, starting from 7 weeks of age and becoming gradually more pronounced. This rise in VEGF-A_164_ protein is not paralleled by an increment of the corresponding transcript, strongly suggesting that it can be sustained by the PKCβII/HuR pathway acting at post-transcriptional level. Nevertheless, the observed increase in HIF-1α at 9 weeks indicates that this transcription factor may favor, in the early phase of the disease, the transcription of other isoforms, possibly neuroprotective, in the attempt to counteract the neurodegenerative effects of VEGF-A_164._ It is worth of note, that our data show a distinct temporal regulation of VEGF-A expression, implicating two different molecular processes: transcriptional and post-transcriptional. In fact, the transcription factor HIF-1α seems to contribute to the earlier increase in VEGF-A protein content, possibly trying to counteract the neurodegenerative effects of DR through the promotion of neuroprotective VEGF-A isoforms, such as VEGF_120/121_. The late rise in VEGF-A seems, instead, to rely upon the PKCβII/HuR cascade acting at post-transcriptional level, which favors the expression of VEGF_164_, the member primary implicated in vascular hyperpermeability and proliferation. To this last regard, these results confirm our previous findings showing, both *in vitro* (Amadio et al., 2008; Amadio et al., 2012; Marchesi et al., 2020; Platania et al., 2020) and *in vivo* (Amadio et al., 2010), a post-transcriptional control of VEGF-A expression mediated by the RNA binding protein (RBP) ELAV/HuR.

In mammals, ELAV proteins are a small family of evolutionarily conserved RBPs, orthologues of the elav gene discovered in the fruit fly Drosophila melanogaster. The family includes the ubiquitously expressed HuR and three neuron-specific members (nELAV), namely HuB, HuC, and HuD. The four ELAV proteins can virtually influence any aspect of the post-synthesis fate of the targeted mRNAs, being stability and translation the most relevant and studied mechanisms. Indeed, following intra- and extracellular inputs, ELAV mainly determine an increase in the cytoplasmic stability and/or rate of translation of the target transcripts, by preferentially binding to ARE (adenine-uracil-rich elements) *cis-acting* elements present within their sequence, although other consensus elements may be implicated (Pascale and Govoni, 2012).

Within this context, in another model of experimental diabetes induced in rodents, namely rats exposed to a single intravenous injection of streptozotocin (STZ), we previously demonstrated that, following a PKC β-mediated phosphorylation, the ELAV/HuR binds to VEGF-A mRNA and positively affects its expression in the retina, thus contributing to abnormally enhanced VEGF-A content in the retinal tissue (Amadio et al., 2010). Notably, STZ-induced diabetic rats show the same features of the NPDR observed in humans, including blood vessels dilation and increased vascular permeability. Further, we also reported that nano-systems loaded with a commercially available siRNA, which specifically switches off the ELAV/HuR expression when injected into the eye of diabetic rats, was able to attenuate the increase in VEGF-A content without suppressing its basal levels (Amadio et al., 2016b). These findings, together with the present data underline the key role of the PKCβ/HuR cascade in regulating the pathologic overexpression of VEGF. Incidentally, the use of nano- or micro-systems could be useful to ameliorate the intra-ocular delivery of pharmacological agents (Conti et al., 1997).

The results obtained by OCT, a non-invasive imaging technique that allows collecting information on the retina morphology, indicate that the retinal nerve fiber layer (RNFL) thickness is dramatically reduced in 46 weeks of age *Ins2* ^*Akita*^ mice, and strongly suggest that this neurodegenerative event may be sustained by the increased VEGF-A_164_ levels. Within this general context, it should be taken into consideration that the VEGF-A gene is alternatively spliced to generate VEGF-A_xxxa_ and VEGF-A_xxxb_ isoforms, being the last ones potentially endowed with anti-angiogenic and anti-permeability properties (Qiu et al., 2009). Of interest, it has been reported that DR is associated with a switch in splicing from anti-towards pro-angiogenic isoforms (Perrin et al., 2005). Although the antibody used in the study allowed us to primarily detect VEGF-A_164a_, it is tempting to speculate that the observed increase in this isoform goes to the detriment of the corresponding VEGF-A_164b_, which has a critical role in cell protection and survival (Peiris-Pagès 2012). Therefore, this switch in isoforms production might promote a degenerative process in the retina leading to a decrease in RNFL thickness, as we detected in the present work. Nevertheless, it should be also underlined that the literature data regarding VEGF-A_xxxb_ isoforms are conflicting, and some authors even question the existence of VEGF-A_xxxb_ isoforms themselves (Harris et al., 2012; Bridgett et al., 2017).

In conclusion, these data seem to suggest that pharmacological intervention in clinical practice might be chosen, among other reasons, based on the specific stages of the diabetic retinopathy to pursue the best clinical outcome. Clinical studies to evaluate this possibility may be warranted.

## Data Availability

The raw data supporting the conclusions of this article will be made available by the authors, without undue reservation.
