# Supplementary figures and images for: Short-and Long-Term Expression of Vegf: A Temporal Regulation of a Key Factor in Diabetic Retinopathy
Source: Front Pharmacol. 2021 Aug 16;12:707909. doi: 10.3389/fphar.2021.707909 (PMC8418071; doi:10.3389/fphar.2021.707909)

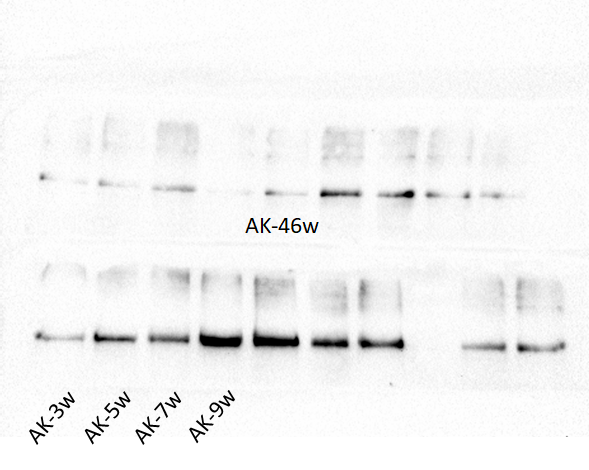

Supplement: Supplementary file 1 [file DataSheet1.ZIP › originals/Original HIF1 alpha fig 4.tif]

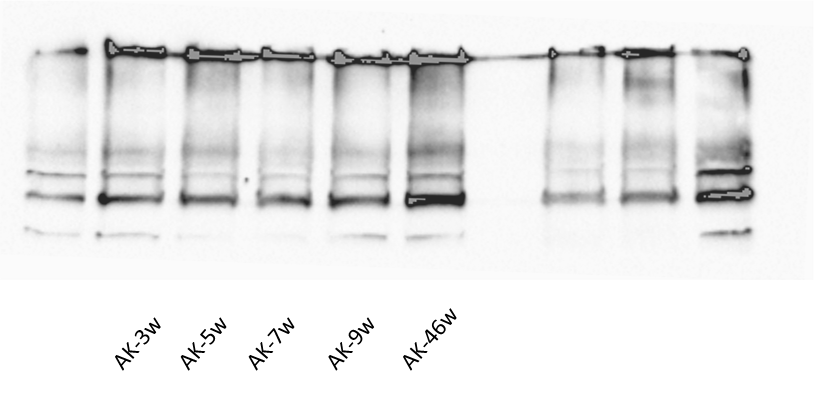

Supplement: Supplementary file 1 [file DataSheet1.ZIP › originals/Original PKC beta fig 5 A.tif]

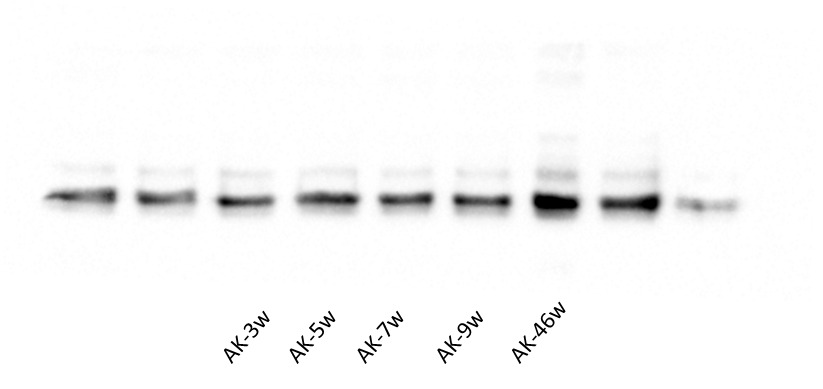

Supplement: Supplementary file 1 [file DataSheet1.ZIP › originals/Original HuR fig 5 B.tif]

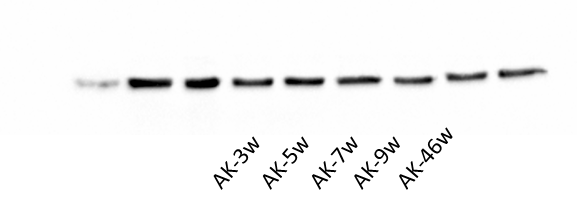

Supplement: Supplementary file 1 [file DataSheet1.ZIP › originals/Original alpha tubulin Fig 3.tif]

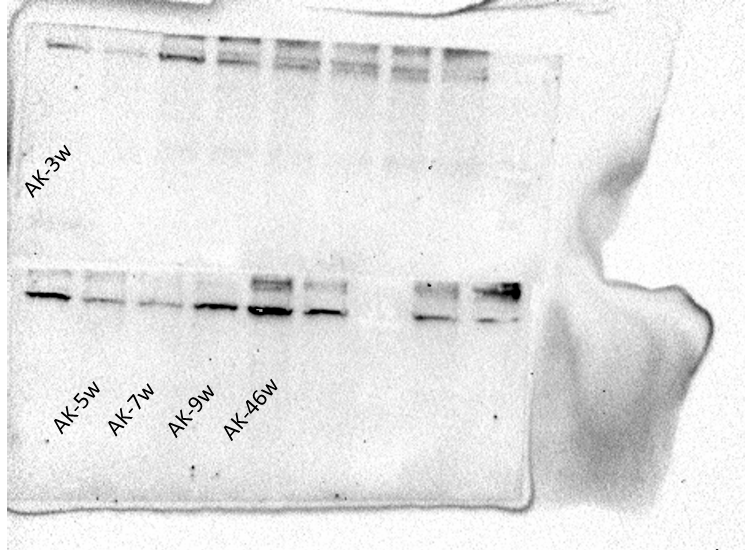

Supplement: Supplementary file 1 [file DataSheet1.ZIP › originals/Original VEGF fig. 3.tif]

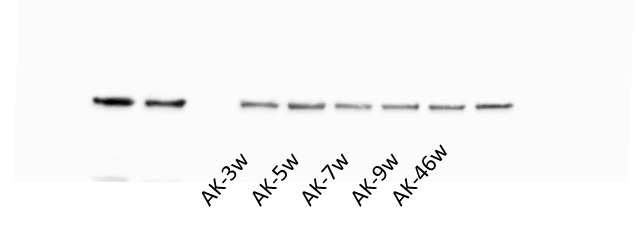

Supplement: Supplementary file 1 [file DataSheet1.ZIP › originals/Original alpha tubulin Fig 5 A.tif]

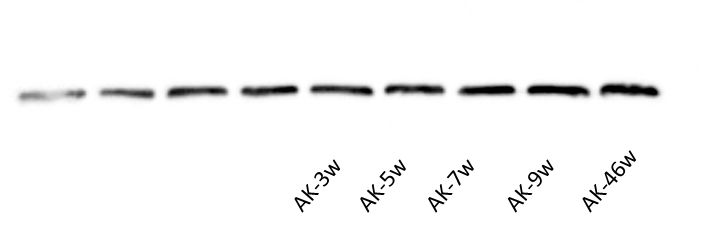

Supplement: Supplementary file 1 [file DataSheet1.ZIP › originals/Original alpha tubulin Fig 5 B.tif]

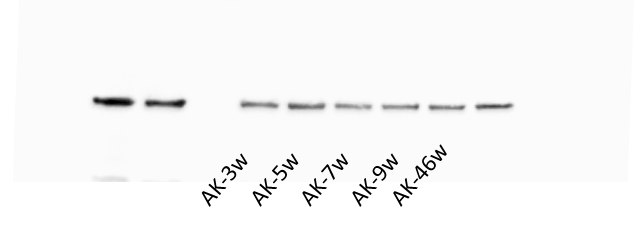

Supplement: Supplementary file 1 [file DataSheet1.ZIP › originals/Original alpha tubulin Fig 4.tif]
